# Evaluation of Moisture Damage in Asphalt Mixtures Under Dynamic Water Pressure Using 3D Laser Scanning

**DOI:** 10.3390/ma19081514

**Published:** 2026-04-09

**Authors:** Wentao Wang, Hua Rong, Yinghao Miao, Linbing Wang

**Affiliations:** 1 National Center for Materials Service Safety, University of Science and Technology Beijing, Beijing 100083, China; wentaowang@ustb.edu.cn (W.W.);; 2 Wuzhou Engineering Group Corporation Ltd., Beijing 100083, China; 3 The Sensing and Perception Lab, School of Environmental, Civil, Agricultural and Mechanical Engineering, University of Georgia, Athens, GA 30602, USA

**Keywords:** asphalt mixture, moisture damage, dynamic pore water pressure, 3D laser scanning technique, erosion mechanism

## Abstract

Under continuous erosion of dynamic water pressure generated by vehicle–water–pavement coupling interaction, asphalt mixture will gradually deteriorate and severe moisture damage finally emerges. The fine aggregate mixture (FAM) component is notably eroded and stripped, while the aggregate component even cracks sometimes. Sufficient attention has not been paid to these critical phenomena. This study employed the 3D laser scanning technique to detect changes in surface roughness of the asphalt mixture before and after it was eroded by dynamic water pressure. The degree of erosion of the asphalt mixture, FAM component, and aggregate component were thereby evaluated. The influences of experimental parameters such as water temperature and pore water pressure magnitude, as well as variable parameters including lithology and asphalt type, were also taken into account. By integrating the detection of physical and mechanical properties evolution of aggregates, the mechanism of moisture damage was comprehensively illustrated from the perspectives of both components of FAM and aggregate. The findings revealed that the 3D laser scanning technique could clearly detect and quantitatively assess the morphological changes on the asphalt mixture surface after been eroded in dynamic water pressure. Both types of asphalt mixtures exhibited varying degrees of erosion and wear, and obvious increases in surface unevenness were observed in each case. Variations in either temperature or pore water pressure magnitude showed limited influence on moisture damage in basalt-based asphalt mixture. In contrast, moisture damage sustained by limestone-based asphalt mixture was notably sensitive to temperature changes but remained largely insensitive to fluctuations in pore water pressure magnitude. The increase in surface roughness of asphalt mixture was primarily attributed to the scouring action of dynamic water pressure, which removed the FAM component surrounding coarse aggregate particles. Degradation in coarse aggregate particles would lead to the deterioration of the entire asphalt mixture. The compatibility between the stripping rate of FAM component and the deterioration rate of coarse aggregate governed the macroscopic manifestation of overall moisture damage in the asphalt mixture.

## 1. Introduction

**Figure 1:**
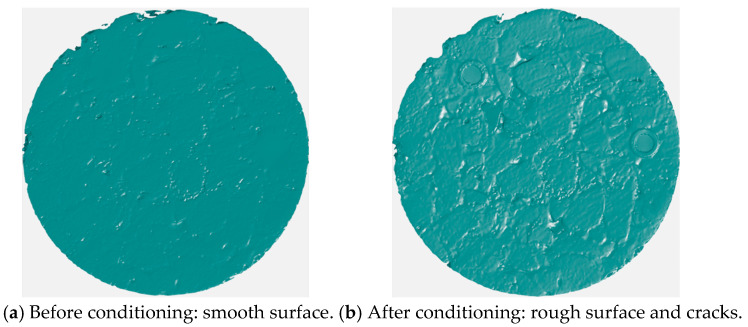
Comparison on asphalt mixture’s surface before and after being conditioned in dynamic water pressure environment.

**Figure 2:**
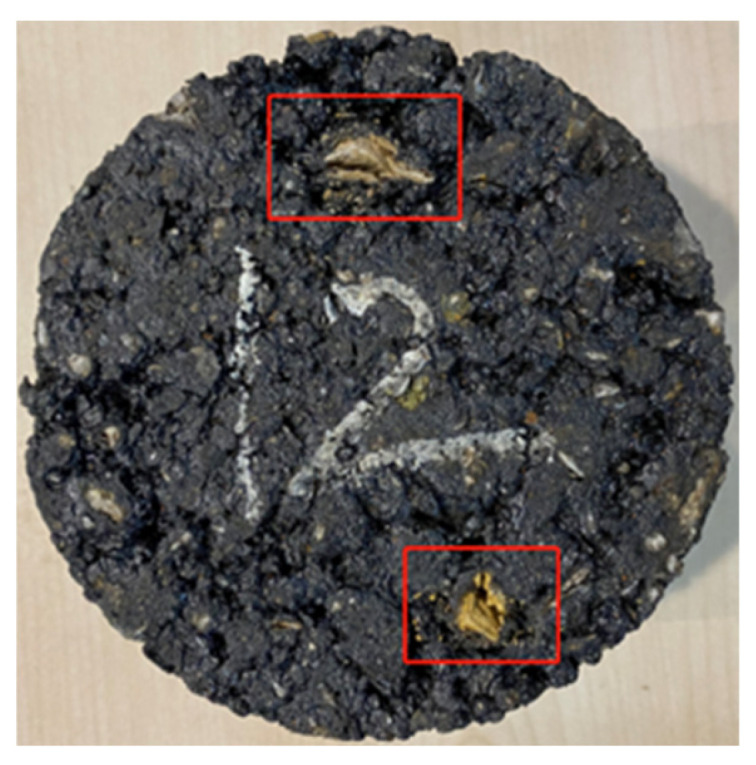
Obvious cracking occurred in aggregate of Marshall specimen after being eroded by dynamic water pressure.

Under continuous erosion of dynamic water pressure generated by vehicle–water–pavement coupling interaction, asphalt mixture will gradually deteriorate and severe moisture damage will finally emerge [1]. Asphalt mixture mainly comprises a coarse aggregate skeleton, fine aggregate mixture (FAM) component, and air void component. The FAM component is notably eroded and stripped away after asphalt mixture is conditioned in a dynamic water pressure environment. Conditioning a sample of asphalt mixture with a polished surface in a dynamic water pressure environment will often observe significant surface roughening, as shown in Figure 1. Obvious cracks sometimes even occurred in aggregate particles of asphalt mixture specimens after being eroded by dynamic water pressure, as shown in Figure 2. This typical phenomenon proved that moisture damage of asphalt mixture related to dynamic water pressure exhibited a totally different damage mechanism compared with damage forms related to traditional water environments like static water immersion and freeze–thaw.

After being eroded by dynamic water pressure, comprehensive performance of the asphalt mixture inevitably deteriorated to a certain extent. Multi-scale testing techniques could be employed to evaluate the degradation characteristics of asphalt material properties. Chen and Huang [2] employed dynamic modulus tests and Superpave indirect tensile tests to evaluate the moisture damage resistance of asphalt mixture specimens subjected to dynamic water pressure. They also assessed the influence of factors such as anti-stripping agents, coarse aggregate angularity, and the number of dynamic water pressure cycles on the test results. Weldegiorgis and Tarefder [3,4] utilized dynamic modulus tests and a newly proposed index to evaluate the moisture damage resistance of specimens subjected to dynamic water pressure and proposed corresponding test procedures. Varveri et al. [5] conducted a comparative study on the differences between long-term and short-term moisture damage using water bath immersion and dynamic water pressure erosion. They found that the former primarily involved moisture diffusion, while the latter was characterized by cyclic pore water pressure, which could effectively accelerate the water environment action process. Tarefder and Ahmad [6] applied dynamic water pressure conditioning to field core specimens and conducted indirect tensile tests for performance evaluation. They found that penetrating and terminal voids had little effect on moisture damage resistance of asphalt mixture. Xu et al. [7] investigated the influence of test parameters and specimen diameter on the accuracy of internal hydraulic characteristic measurements in asphalt mixture specimens after dynamic water scour, using relative error indicators to identify the optimal combination of test parameters that maximize image resolution and contrast. Varveri et al. [8] employed CT scanning and image analysis techniques to analyze the damage mechanisms induced by dynamic water pressure. Their study revealed that strength degradation was related to the state and duration of dynamic water pressure action, as well as the type of raw materials. Image analysis techniques visually revealed the mechanism by which dynamic water pressure affected air void content and connectivity inside asphalt mixture. Faisal et al. [9] conducted tests on asphalt mixture specimens subjected to dynamic water conditioning and found that the modulus of FAM decreased by 60% compared to dry conditions after water action, which led to a 70% reduction in the overall indentation modulus of asphalt mixture. Khorasani and Al-Rub [10] employed nanoindentation to investigate the local nanoscale mechanical properties of the FAM, aggregate, and their interaction zone within asphalt mixture. They found that the modulus and hardness values of the interaction zone fell between those of the aggregate and FAM. Yao et al. [11] utilized atomic force microscope (AFM) and nanoindentation to quantitatively determined the strength loss and damage types (adhesive or cohesive failure) of asphalt materials after moisture damage, and validated the nanoscale test results through surface energy techniques and freeze–thaw splitting tests.

Based on the above research, it is evident that employing a variety of testing techniques could effectively characterize the multi-scale performance degradation patterns of asphalt-related materials subjected to moisture damage. These studies exhibited the following characteristics: Firstly, the associated testing costs were relatively high, particularly for micro- and nano-scale techniques such as CT and AFM. Systematic testing of a large number of moisture-damaged asphalt-material specimens required careful consideration of the experimental expenses beforehand. Secondly, many of these tests were destructive, such as mechanical strength and dynamic modulus tests, making it difficult to characterize the performance degradation of the same specimen before and after moisture damage. Thirdly, there was a lack of standardized image-based methods for directly observing and analyzing the morphological changes associated with the damage and deterioration of asphalt mixture specimens. Direct photography could capture apparent changes on the specimen surface, as illustrated in Figure 1 and Figure 2. However, more precise quantitative analysis of surface variations required the use of more sophisticated techniques. CT technology could effectively acquire images of internal damage characteristics within asphalt mixtures [12,13,14]. Considering that the erosive effect of dynamic water pressure on an asphalt mixture specimen was a dynamic process progressing from the surface inward, CT scanning was highly effective for characterizing changes in internal pore channels after a certain degree of internal damage had developed. Nevertheless, its ability to assess morphological changes on the specimen surface during the initial stage of damage was limited. 3D laser scanning technology, on the other hand, could effectively measure and quantify the roughness characteristics of the macroscopic surface texture of asphalt pavements [15,16]. It was particularly well-suited for the quantitative analysis of surface roughening induced by erosion and scouring from dynamic water pressure on asphalt mixture.

While the aforementioned studies primarily focused on the overall performance changes exhibited by entire asphalt mixture, several critical phenomena associated with the stepwise erosion process of FAM component induced by dynamic water pressure, as well as the direct development of visible cracks in coarse aggregate particles, have not received adequate attention and investigation. The observations of Figure 1 and Figure 2 strongly demonstrated the importance of fundamental components such as the FAM component and coarse aggregate particles in determining the overall moisture damage resistance of an asphalt mixture. Furthermore, compared with conducting complicated mechanical tests to evaluate changes in the comprehensive properties of eroded asphalt mixture, it is more straightforward and effective to directly observe and assess its surface erosion changes, which can be well achieved by the 3D laser scanning technique.

This study employed the 3D laser scanning technique to detect changes in surface roughness of asphalt mixture before and after it was eroded by dynamic water pressure. The degree of erosion of the asphalt mixture, FAM component, and aggregate component were thereby evaluated. The influence of evaluating dimension on surface erosion degree was discussed. The impacts of experimental parameters such as water temperature and pore water pressure magnitude, as well as variable parameters including lithology and asphalt type, were also taken into account. By integrating the detection of physical and mechanical properties evolution of aggregates, the mechanism of moisture damage was comprehensively illustrated from the perspectives of both components of FAM and aggregate.

## 2. Materials and Sample Preparation

**Figure 3:**
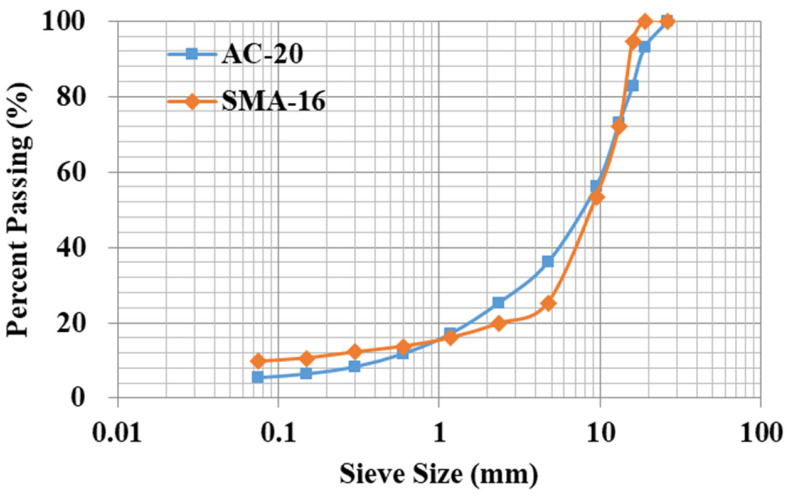
Two types of aggregate gradations.

In this study, two types of typical asphalt mixture samples were fabricated for comprehensive evaluation. A base asphalt binder labeled 70# and a styrene–butadiene–styrene (SBS)-modified asphalt binder were selected. The asphalt binder samples’ physical properties were inspected, and their testing results are summarized in Table 1, both of which satisfied the technical requirements of the specification [17]. Limestone and basalt aggregate were adopted with two different gradations of AC-20 and SMA-16 respectively, as shown in Figure 3. AC-20 and SMA-16 were usually applied to construct the middle layer and top surface layer of asphalt pavement, respectively. Therefore, these two types of typical asphalt mixture were selected to comparatively evaluate moisture damage resistance induced by dynamic pore water pressure. For AC-20, the 70# base asphalt binder was applied to fabricate hot mix asphalt (HMA) samples with the asphalt content of 3.94% and the air void content of 4.7%. For SMA-16, the SBS-modified asphalt binder was used to prepare asphalt mixture samples with the asphalt content of 5.57%, the fiber content of 0.3%, and the air void content of 3.8%. Specifically, the lithology of the fine aggregate in SMA-16 was limestone. Basic service properties of asphalt mixture samples were tested, as shown in Table 2, both of which met the requirements of the specification [17].

**Figure 4:**
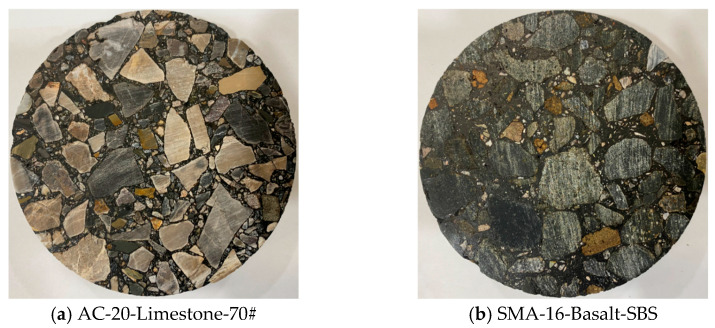
Typical examples of fabricated asphalt mixture samples.

Asphalt mixture samples were fabricated at relevant suitable temperatures according to the requirements of the specification [18]. The mixing temperatures for AC-20 and SMA-16 were 160 °C and 170 °C respectively, and their compacting temperatures were 150 °C and 160 °C individually. The Superpave Gyratory Compactor (SGC) device (Controls, Aosta, Italy) was applied to compact specimens of asphalt mixture. The cylindrical sample’s dimensions were 150 mm in diameter and 150 mm in height. The core drilling machine (Cayken, Shanghai, China) and the auto saw device (Zhongdeshenke, Beijing, China) were applied to extract a core sample with a uniform distribution of air voids from a SGC specimen, while the dimensions of the core sample were 100 mm in diameter and 100 mm in height. The core specimen of the asphalt mixture was further divided uniformly into three pieces along the height direction, and thus the dimensions of the final sample of asphalt mixture were 100 mm in diameter and 30 mm in height. The circular cross-sections of the asphalt mixture sample’s top and bottom sides were polished successively using 100-, 240-, 400-, 600-, 800-, 1000-, 1200-, and 1500-grid sandpapers. A smooth cross-section with enough representative coarse aggregates was selected for further erosion by different dynamic pore water pressure environments. Typical examples of fabricated asphalt mixture samples for AC-20-Limestone-70# and SMA-16-Basalt-SBS are shown in Figure 4. Each group of dynamic water pressure conditioning was prepared with one HMA specimen.

## 3. Test and Analysis Methods

### 3.1. Dynamic Pore Water Pressure Conditioning Method

The Moisture Induced Sensitivity Tester (MIST) device (Hangtianhangyu, Beijing, China) was applied to provide dynamic pore water pressure environment for stripping effect on the asphalt mixture and aggregate. This kind of dynamic water was formed by the repeat process of inflation and restitution of a bladder inside the device [19]. Three variable parameters could be adjusted to simulate different dynamic water environments, which included water temperature, magnitude of pore water pressure, and conditioning duration. In this study, the parameter of conditioning duration was fixed at 4000 cycles for every water-conditioning environment, while the influential degrees of water temperature and pore water pressure magnitude were evaluated at three levels respectively. Five environments of dynamic pore water pressure were taken into account in this study, and the proposal was summarized in Table 3. In particular, the expression of 60 °C—0.4 MPa represented that a sample of asphalt mixture or aggregate was conditioned in a dynamic water environment for 4000 cycles, in which the water temperature was 60 °C and the pore water pressure magnitude was 0.4 MPa.

### 3.2. The 3D Laser Scanning Technique

**Figure 5:**
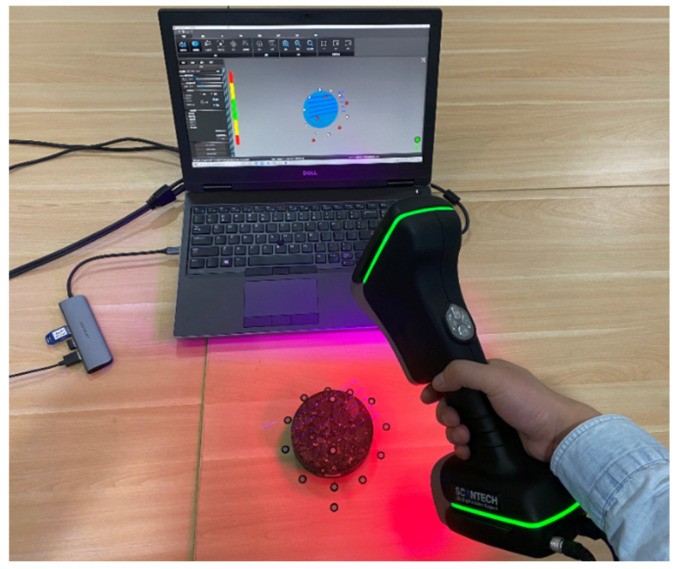
Measuring a sample’s surface roughness using the 3D laser scanning technique.

The 3D laser scanning technique was applied to measure the surface roughness of asphalt mixture samples, as shown in Figure 5. A sample was placed on the horizontal test bench with a series of reflective marking points being pasted around the sample and on its surface. As the FAM component was easily eroded and scoured away by dynamic water pressure, the pasted place of the reflective marking points on the sample’s surface was chosen within coarse aggregate particles. These scan-blank points on the sample’s surface would be auto-filled by the algorithm during the analysis process. The obtained points were presented by x-y-z coordinates, and the scanning accuracy was set at 0.1 mm.

**Figure 6:**
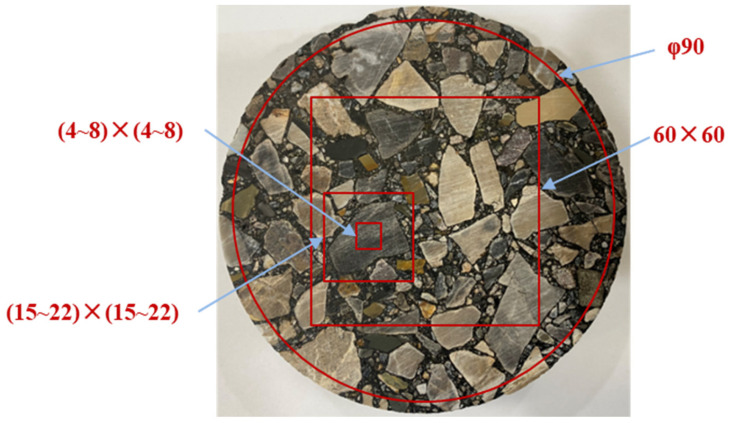
Different evaluating dimensions during 3D laser scanning (Unit: mm).

The edge of the asphalt mixture sample might be deformed and become irregular due to stripping. Therefore, the central circular cross-section with the 90 mm diameter was selected from the sample’s 100 mm diameter range to ensure uniformity and integrity of 3D scanning data distribution. The variation in surface roughness for asphalt mixture could be evaluated based on the measured 3D scanning data from this 90 mm diameter central circular cross-section. In addition, the central square cross-section with 60 mm side length was also chosen from the sample surface to compare the influence of evaluating dimension on roughness assessment. Two representative coarse aggregate particles with their surrounded FAM component for each group were selected for further analysis and discussion. To evaluate changes in the interface between the aggregate and FAM component, a rectangle cross-section with a 15~22 mm side-length range was selected for 3D scanning data analysis. The rectangle cross-section with a 4~8 mm side-length range was further determined within the chosen representative coarse aggregate particle to discuss changes in aggregate surface roughness. Different evaluating dimensions for surface erosion degree analysis based on 3D scanning are shown in Figure 6.

Both statistical, fractal, and entropy indicators were calculated based on the laser-scanned data of each point within the selected cross-section region mentioned above to analyze surface texture variation in asphalt mixture samples [20,21,22]. The statistical indicators of *R_a_* and *R_q_* were effective to evaluate surface roughness of asphalt mixture, and were thus selected in this study to characterize texture elevation values. *R_a_* represented the arithmetic mean deviation of asphalt mixture surface points’ elevations from the mean elevation within the selected region. *R_q_* quantified the root mean square deviation of asphalt mixture surface points’ elevations relative to the mean elevation across the analyzed region. *R_a_* and *R_q_* were determined according to the Formulas (1) and (2), in which *R_c_* was the mean elevation within the selected region and *z_i_* was each point’s elevation.

(1) $$R_{a}=\frac{1}{n}\sum_{i=1}^{n}\left|z_{i}-R_{c}\right|$$

(2) $$R_{q}=\sqrt{\frac{1}{n-1}\sum_{i=1}^{n}{\left(z_{i}-R_{c}\right)}^{2}}$$

The fractal dimension *D* was a scaling exponent that quantitatively characterized the roughness or structural complexity of the sample’s surface. *D* reflected the non-integer dimensional properties of the sample’s morphology as a function of measurement scale, and the detailed description can be referred to in the previous study [21]. Entropy *E* was a thermodynamic state function that quantified the degree of disorder or randomness in a system; in this study, it meant the uncertainty or average information content of a random variable. *E* was determined based on the Formula (3), in which *p_i_* was the probability of grey level *i* and *N_g_* meant an image had the maximum grey level. Detailed illustration of *E* can be found in the previous study [22].

(3) $$E=\sum_{i=1}^{N_{g}}p_{i}\log_{2}(\frac{1}{p_{i}})$$

### 3.3. Aggregate Crushing Value Testing Method

**Figure 7:**
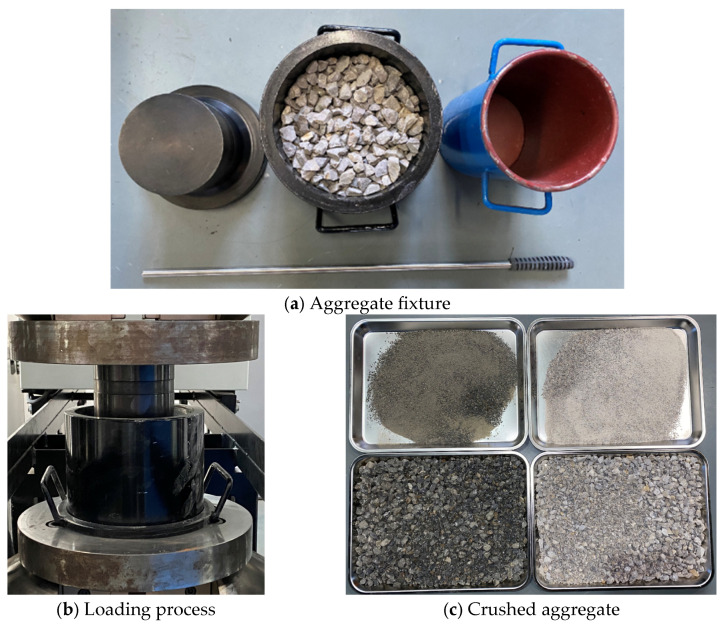
Aggregate crushing value test.

For limestone and basalt, aggregates with particle size within 9.5 mm–13.2 mm were cleaned firstly and then conditioned in dynamic water pressure environments. Each group contained a mass of about 3000 g, and two parallel samples were considered. The physical properties of mass loss and water absorption rate for aggregate samples before and after being conditioned were evaluated, while the mechanical property of crushing value was assessed. In the aggregate crushing value test, aggregate particles were firstly poured into a fixture and then evenly applied by a gradual increasing load using a Material Testing System (MTS). The loading procedure should be as follows: ramped the force to 400 kN over a period of 10 min, held the constant load for 5 s, and subsequently performed unloading. The crushed aggregate sample was then sieved using the 2.36 mm sieve. The process of the aggregate crushing value test is shown in Figure 7. The crushing value was defined as the ratio of the mass passing the 2.36 mm sieve to the total mass of the crushed aggregate sample. A higher crushing value indicated weaker mechanical performance of the aggregate in this study.

## 4. Results and Discussions

### 4.1. Surface Erosion Degree Based on 3D Scanning

#### 4.1.1. Evaluating Dimension: Asphalt Mixture Specimen

After being conditioned in dynamic water pressure environments with different contents of severity, the FAM component on the surface of an asphalt mixture specimen was eroded and scoured away. The surfaces of coarse aggregate particles also endured the erosion effects. Therefore, these eroding actions would alter the surface texture of an asphalt mixture specimen and make its surface rougher. The 3D laser scanning technology was adopted to acquire elevation data of testing points spaced at 0.1 mm intervals on the specimen surface. By selecting an appropriate cross-sectional area, the changes in surface roughness of a sample before and after erosion within this region could be calculated and characterized. The erosion degree of the sample within the selected region could thus be evaluated.

**Figure 8:**
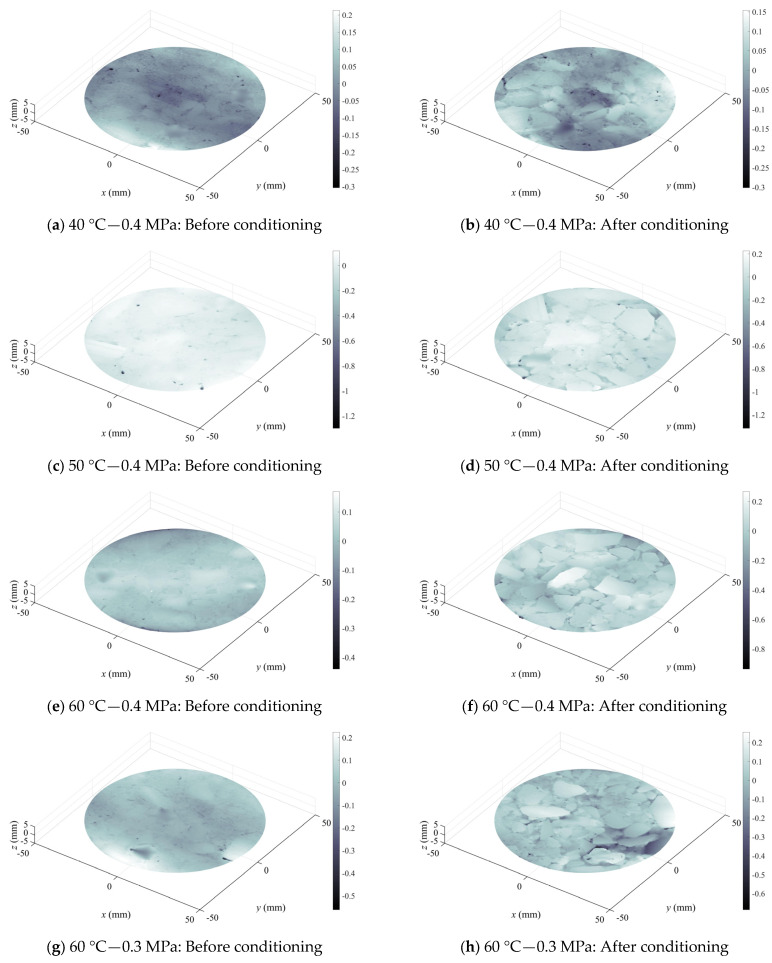

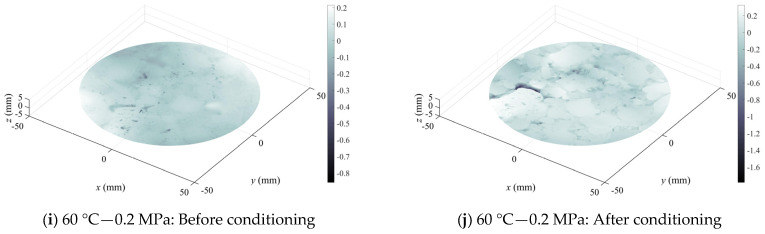
Variation in surface roughness AC-20-Limestone-70# HMA samples before and after being conditioned in dynamic water pressure environment.

**Figure 9:**
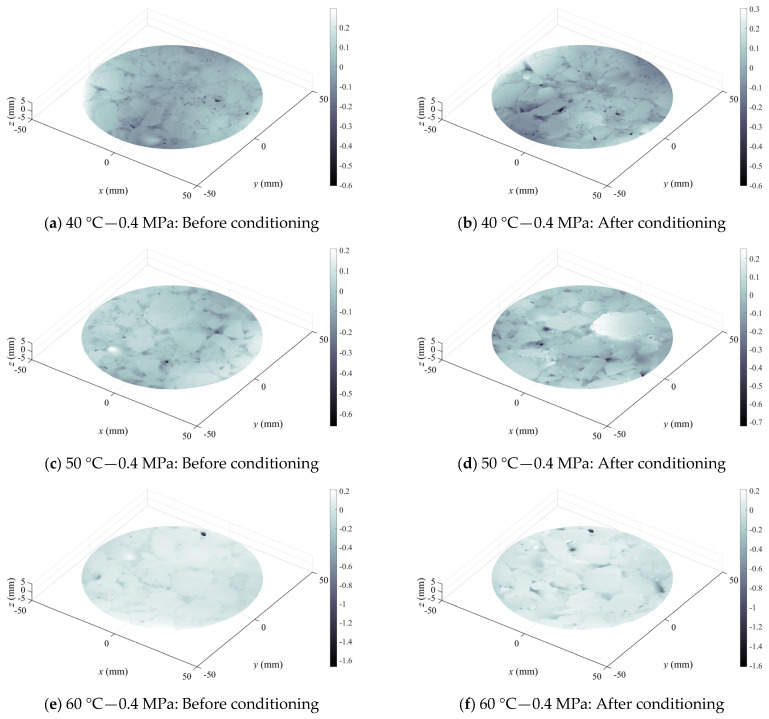

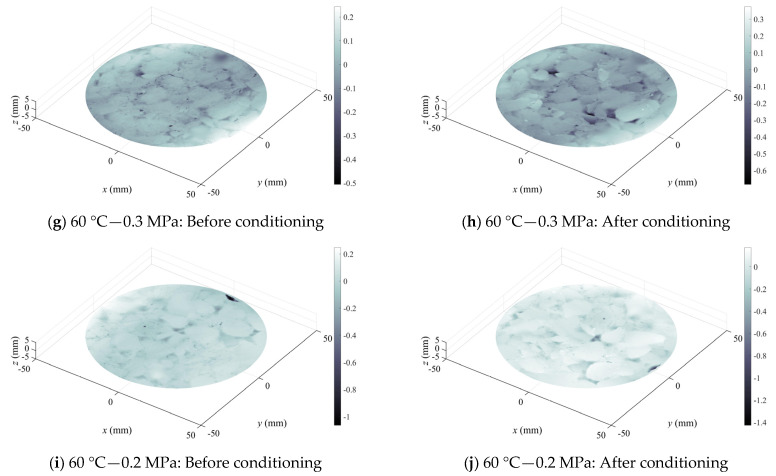
Variation in surface roughness SMA-16-Basalt-SBS HMA samples before and after being conditioned in dynamic water pressure environment.

Figure 8 and Figure 9 illustrated surface texture variations within a 90 mm diameter circular cross-section at the center of specimens for two typical asphalt mixtures, AC-20-Limestone-70# and SMA-16-Basalt-SBS, before and after erosion, respectively. It could be observed that the outer contours of coarse aggregate particles on the HMA specimen surface became distinctly visible after erosion under various conditions of dynamic water pressure. The phenomena were attributed to the erosion and significant stripping of the FAM component filling within the coarse aggregate skeleton by dynamic water pressure. The areas between coarse aggregate particles which were the locations of the FAM component showed dark color. After stripping, the dark coloration would be intensified, as shown in Figure 8a,b compared to Figure 9c,d. Alternatively, the number of dark areas increased markedly, as shown in Figure 8c,d versus Figure 9a,b. In other cases, originally isolated small dark areas might coalesce into a continuous larger dark region following erosion, as demonstrated in Figure 8g,h compared to Figure 9g,h. These observations indicated that dynamic water pressure induced significant erosion effects on both AC-20-Limestone-70# and SMA-16-Basalt-SBS asphalt mixture specimens. Comparing two types of asphalt mixture after stripping, it revealed that the AC-20-Limestone-70# specimens exhibited larger continuous dark areas between coarse aggregate particles, e.g., Figure 8b,h, whereas the SMA-16-Basalt-SBS specimens predominantly displayed scattered and isolated dark areas, e.g., Figure 9h. This finding also suggested that the resistance of the FAM component to erosion by dynamic water pressure was weaker for the base 70# asphalt binder compared to the SBS-modified asphalt binder.

**Figure 10:**
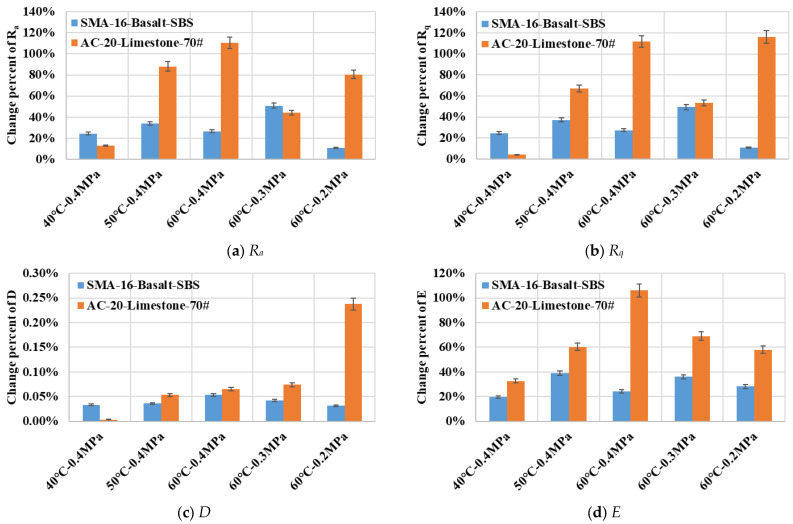
Variations in surface roughness indicators for HMA samples with different severities of dynamic water pressure.

After being eroded by dynamic water pressure, the surface roughness indicators of both types of asphalt mixture specimens exhibited significant changes, as shown in Figure 10 with percentage error bars. Figure 10a,b reveals that statistical indicators, including the arithmetic mean deviation *R_a_* and the root mean square deviation *R_q_*, increased across all experimental groups. It indicated an obvious rise in surface unevenness of all HMA specimens. However, an opposite trend was observed under the 40 °C water condition. This might be attributed to the relatively mild environment of the 40 °C water temperature in this study, resulting in no significant difference in erosion degrees between the two types of asphalt mixture. When the water pressure magnitude was maintained at 0.4 MPa, the change rate in roughness indicators for the limestone-based asphalt mixture gradually increased with a rising water temperature. It suggested that the damage degree sustained by the limestone-based asphalt mixture under dynamic water pressure erosion intensified with increasing water temperature. Under the harsh conditions of 60 °C water temperature, an increase in water pressure magnitude did not induce a clear change in the erosion degree of the limestone-based asphalt mixture. This indicated that the erosion effect of water pressure magnitude on a HMA specimen might be related to the distribution of its various components on the contact surface.

Figure 10c,d demonstrates that indicators such as the fractal dimension *D* and the entropy *E* increased for all experimental groups after being eroded by dynamic water pressure. For the limestone-based asphalt mixture, the fractal dimension *D* increased with a rising water temperature but decreased with an increasing water pressure magnitude. Furthermore, the entropy *E* for the limestone-based asphalt mixture also increased with a rising water temperature, while it exhibited an increasing trend with the elevation of water pressure magnitude. The overall variation in the fractal dimension *D* for the basalt-based asphalt mixture was relatively small. It also increased slowly with a rising water temperature, but gradually increased with the magnitude of water pressure, while the later phenomenon was converse to that of the limestone-based asphalt mixture. The changes in the entropy *E* for the basalt-based asphalt mixture did not show a clear pattern with variations in parameters such as water temperature and water pressure magnitude.

All roughness indicators for the limestone-based asphalt mixture increased significantly with a rising water temperature, whereas the changes in surface roughness indicators for the basalt-based asphalt mixture did not exhibit a clear pattern with dynamic water pressure conditions. Compared to the statistical indices, parameters such as fractal dimension *D* and entropy *E* could better reflect the fluctuating pattern of asphalt mixture’s erosion degree with changes in water pressure magnitude. Overall, the change degree in roughness indices for the limestone-based asphalt mixture exceeded that of the basalt-based asphalt mixture. It indicated that the basalt-based asphalt mixture possessed relative superior resistance to erosion by dynamic water pressure. This phenomenon was closely related to the types of asphalt binder used in the fabrication of asphalt mixture specimens. Although the basalt-based asphalt mixture was also eroded under different water pressure environments, the fluctuations in its surface roughness indices did not display a clear pattern and were relatively small in overall magnitude. It implied that the basalt-based asphalt mixture exhibited more stable service performance in a dynamic water pressure environment compared to the limestone-based asphalt mixture.

#### 4.1.2. Evaluating Dimension: Aggregate Considering the FAM Component Interface

**Figure 11:**
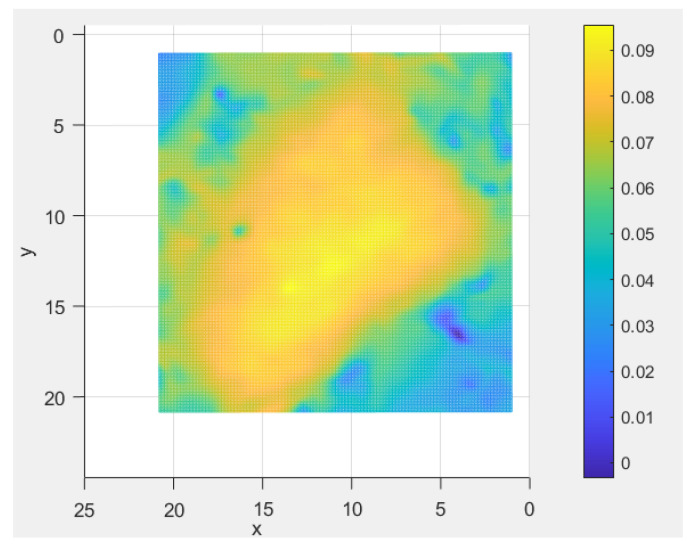
A typical coarse aggregate particle with surrounded FAM component.

The asphalt mixture of AC-20-Limestone-70# was characterized as a suspension-dense structure, wherein the limestone coarse aggregate particles were encapsulated by the FAM component and air void component. In contrast, the asphalt mixture of SMA-16-Basalt-SBS was featured as a stone-on-stone skeleton interlocking structure, wherein the basalt coarse aggregate particles were also surrounded by the FAM component and air void component, but these particles were additionally in direct contact and interlock with one another. An extracted image depicting a single coarse aggregate particle along with its surrounded FAM component was presented in Figure 11. This allowed for direct observation of the erosion characteristics of the fundamental constituent components of asphalt mixture under the erosion of dynamic water pressure. Although the aforementioned analysis also pertained to the air void component, the 3D laser scanning technique employed in this study could not precisely identify microscopic air voids. Therefore, the air void component of asphalt mixture was collectively incorporated into the FAM component for a holistic analysis.

**Figure 12:**
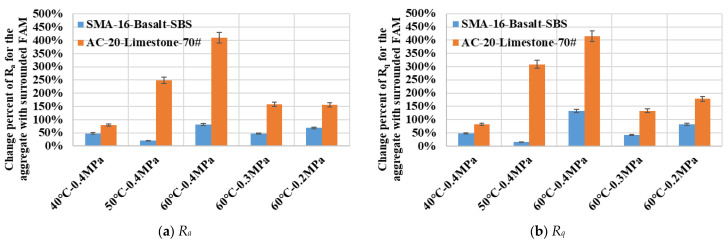
Variations in surface roughness indicators for aggregates and surrounded FAM components with different severities of dynamic water pressure.

Figure 12 illustrates the variations in surface roughness indices for coarse aggregate particles and their surrounded FAM components with erosion under different dynamic water pressure conditions. It could be observed that after being eroded in dynamic water pressure, statistical indicators such as the arithmetic mean deviation *R_a_* and the root mean square deviation *R_q_* for both types of asphalt mixture increased significantly. It indicated a rise in surface unevenness within the dimensional scale of the coarse aggregate particles and their surrounded FAM components. For the limestone-based FAM component, *R_a_* and *R_q_* exhibited clear increasing trends with a rising water temperature, but their variations with water pressure magnitude were less systematic. The increases in *R_a_* and *R_q_* for the basalt-based FAM component were considerably smaller than those observed for the limestone-based FAM component. Furthermore, the magnitude of these increases did not fluctuate significantly across different dynamic water pressure conditions. It suggested that the service behavior of the basalt-based FAM component was relatively stable. The greatest increase in roughness indices for both types of asphalt mixture occurred in the most severe dynamic water pressure condition (60 °C—0.4 MPa). Figure 12 clearly presented the stability characteristics of the base 70# asphalt mortar and the SBS-modified asphalt mortar when subjected to the erosion by dynamic water pressure.

#### 4.1.3. Evaluating Dimension: Aggregate

Under different service environments, coarse aggregate particles in asphalt mixture were conventionally considered to function primarily in bearing vehicle loads. However, these particles themselves were also susceptible to performance degradation due to environmental influences. In this study, suitable coarse aggregate particles were selected from cross-sections of asphalt mixture specimens, and small local areas within these particles were chosen for further analysis. Utilizing data acquired via 3D laser scanning, the evolution of surface roughness characteristics of coarse aggregate particles before and after erosion by dynamic water pressure was investigated.

**Figure 13:**
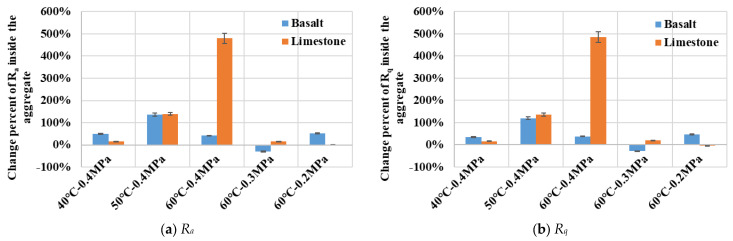
Variations in surface roughness indicators for aggregates with different severities of dynamic water pressure.

As illustrated in Figure 13, the surface roughness of limestone exhibited a clear increasing trend with a rising water temperature. The most pronounced increase in surface roughness after erosion occurred under the most severe dynamic water pressure condition of 60 °C and 0.4 MPa. In contrast, the change percent in surface roughness for basalt subjected to dynamic water pressure was smaller than that observed for limestone. Furthermore, the change percent for basalt showed a greater dependency on variations in water temperature than on the magnitude of dynamic water pressure. Under the dynamic water pressure environment of 50 °C and 0.4 MPa, the surface roughness of both limestone and basalt was noticeably affected by dynamic water erosion. Notably, a decrease in surface roughness was observed for both rock types, with basalt exhibiting a relatively more pronounced reduction. This phenomenon might be attributed to the presence of micropores or microcracks on the coarse aggregate surfaces. Under the sustained erosion of dynamic water pressure, these superficial imperfections underwent continuous abrasion, leading to the detachment of finer particles and resulting in a relatively smoother surface. Additionally, the continuous erosion and stripping of the FAM component surrounding the coarse aggregate particles might induce displacement and rotation of the coarse aggregate particles within asphalt mixture. This could contribute to a relative decrease in the roughness values derived from the 3D laser scanning data of the eroded aggregate surfaces.

#### 4.1.4. Influence of Evaluating Dimension on Surface Erosion Degree

The values of the arithmetic mean deviation *R_a_* and the root mean square deviation *R_q_* presented in Figure 10 represent the roughness of the entire cross-section of asphalt mixture, which essentially integrated the areas encompassing multiple individual coarse aggregate particles and their surrounded FAM components shown in Figure 12. It could be observed that the trends in the indicators of *R_a_* and *R_q_* for AC-20-Limestone-70# with varying parameters of dynamic water pressure are broadly consistent between Figure 10 and Figure 12. However, differing trends were noted for the indicators of *R_a_* and *R_q_* of SMA-16-Basalt-SBS between these two figures. This suggested that for a base 70# asphalt mortar FAM, which was susceptible to erosion by dynamic water pressure, the region comprising a single coarse aggregate particle and its surrounded FAM component could basically characterize the degree of stripping affecting the asphalt mixture specimen. Selecting a small evaluation area encompassing several coarse aggregate particles appeared sufficient to effectively represent the overall roughness change characteristics of the entire asphalt mixture. Conversely, for an SBS-modified asphalt mortar FAM that exhibited good resistance to the erosion of dynamic water pressure, a larger evaluation area was necessary to effectively characterize the overall roughness change in the entire asphalt mixture.

The *R_a_* and *R_q_* values in Figure 12 quantify the roughness within the region encompassing a single coarse aggregate particle and its surrounded FAM component. This region included the smaller intra-particle area analyzed in Figure 13. Although the percentage changes in the indices shown in Figure 13 are relatively large, the absolute magnitudes of these indices are considerably smaller than those presented in Figure 12. A combined examination of Figure 12 and Figure 13 reveals that, within the evaluation scale of a single coarse aggregate particle and its surrounded FAM component, the change degree of roughness originating from the intra-aggregate region is substantially less than the roughness increase caused by the erosion and denudation of the FAM component. Consequently, under the erosion duration of 4000 cycles of dynamic water pressure applied in this study, although some surface erosion and roughening of the coarse aggregate particles occurred, the erosion and stripping effect on the FAM component within the specimen cross-section was significantly more pronounced. A further increase in the erosion duration would likely induce more extensive erosion of coarse aggregate particles, potentially revealing more substantial changes in their surface roughness.

### 4.2. Physical and Mechanical Properties of Aggregates

**Figure 14:**
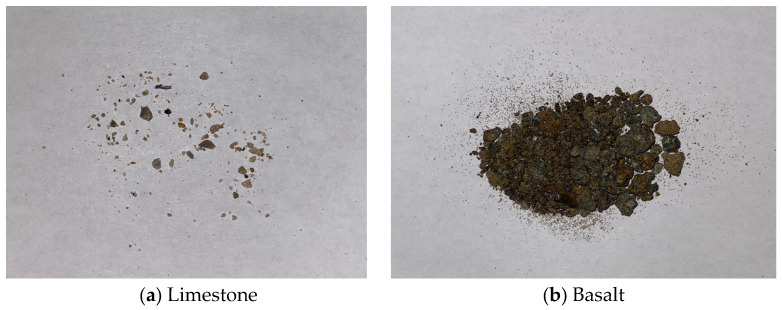
Small particles scoured away from coarse aggregates after being conditioned in dynamic water pressure.

**Figure 15:**
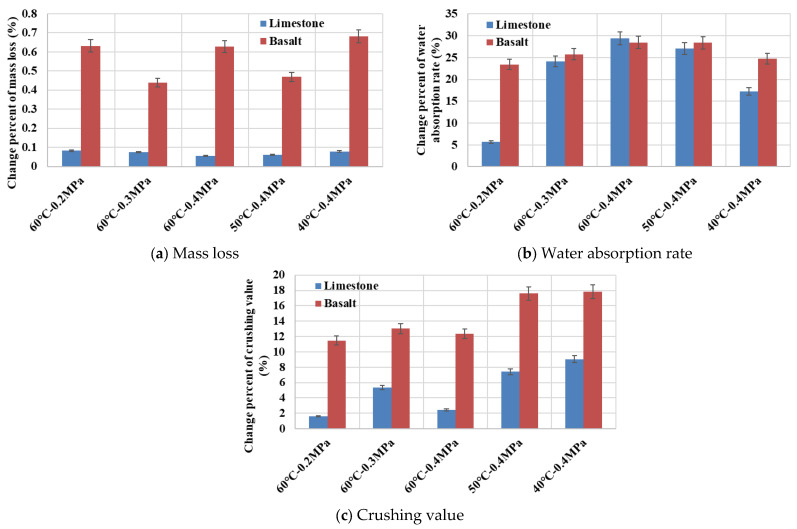
Physical and mechanical properties of aggregates.

Coarse aggregates of limestone and basalt both were conditioned in dynamic water pressure environments, and their physical and mechanical properties were evaluated. Figure 14 shows the small granular debris detached from limestone and basalt aggregates after being subjected to erosion in a dynamic water pressure environment of 60 °C and 0.4 MPa. It was clearly observed that the number of debris detached from limestone was relatively small, whereas basalt shed considerably more debris. This phenomenon was consistently observed following erosion under various dynamic water pressure environments. As illustrated in Figure 15a, the mass loss of limestone was typically around 2 g, demonstrating relative stability and greater resistance to erosion by dynamic water pressure. In contrast, the mass loss of basalt approached 20 g, with substantial variation between different experimental groups. This variability might be attributed to the presence of surface imperfections such as microcracks or micropores on the aggregate particles. Erosion by dynamic water could lead to the detachment of larger particles from these flawed areas, resulting in the observed randomness and lack of a clear trend in basalt mass loss. The phenomenon of basalt shedding more granular debris after erosion provided indirect support for the explanation regarding the decrease in basalt surface roughness.

As shown in Figure 15b, the percentage change in aggregate water absorption rate was influenced by erosion in a dynamic water pressure environment. Basalt exhibited a higher absolute water absorption rate value compared to limestone, although the overall range of variation was not substantial. The water absorption rate of limestone varied between 0.04% and 0.17%, while that of basalt ranged from 0.37% to 0.42%. For both aggregate types, water absorption rate increased with greater severity of water temperature and dynamic water pressure magnitude, with temperature exerting a more pronounced influence than water pressure magnitude [23]. The increase in aggregate water absorption rate might be attributed to the erosion and detachment of surface debris under dynamic water pressure conditions, leading to a slight increase in surface porosity.

Figure 15c illustrated the percentage change in aggregate crushing value resulting from erosion in dynamic water pressure. Under identical test conditions, the absolute crushing value of basalt was 3.5% to 4.5% lower than that of limestone, and it indicated that basalt possessed superior mechanical properties. However, the extent of change in the crushing value was generally smaller for limestone than for basalt, and it suggested that limestone exhibited greater stability under dynamic water erosion. The influence of water temperature variations on the crushing value was consistently more significant than that of water pressure magnitude variations [23], with limestone showing an increase of 0.24% to 1.35% and basalt showing an increase of 1.22% to 1.89%. Notably, as the severity of the dynamic water pressure intensified, the aggregate crushing value tended to decrease. This phenomenon might be attributed to the detachment of weaker surface debris from aggregate particles, leaving behind a more robust and resilient remaining structure. In summary, while the basalt selected for this study demonstrated superior mechanical properties compared to limestone, it possessed a greater abundance of surface micropores or microcracks. This characteristic made the physical and mechanical performance of basalt more susceptible to fluctuations under the erosive action of dynamic water pressure.

### 4.3. Discussion on Moisture Damage Mechanism of Asphalt Mixture

Moisture damage in asphalt mixtures induced by erosion from dynamic water pressure was typically accompanied by significant stripping of the asphalt mortar FAM component. Following the conclusion of experiments, a substantial quantity of eroded fine aggregate was commonly observed within the vessels of environmental simulation equipment. The moisture damage mechanism exhibited by this characteristic phenomenon was fundamentally distinct from that associated with traditional water environments, such as static water immersion or freeze–thaw conditions.

When an asphalt mixture specimen was placed in a dynamic water pressure environment, the asphalt binder endured the erosion action of coupled multi-physical fields. This progressively reduced its internal cohesive energy and its adhesive energy with the aggregate. Consequently, cracks initiated either within the asphalt mastic or at the asphalt–aggregate interface. The adhesion between the asphalt binder and fine aggregate weakened, which led to stripping, and even small fragments of the asphalt mortar FAM could be directly eroded away. This manifested globally as the pronounced stripping of the asphalt mortar FAM component within an asphalt mixture. The coarse aggregate component inside the asphalt mixture was also subject to erosion. Micropores or microcracks on the surfaces of coarse aggregate particles would serve as initiation points for erosion by dynamic water pressure, and it would result in the detachment of weak surface layers. The morphological characteristics of the coarse aggregate particles were altered, such as surface roughness, and their physical and mechanical properties correspondingly deteriorated.

The variation in surface roughness indicators of asphalt mixture samples in this study directly reflected the stripping degrees of asphalt-material components. A larger value of surface roughness indicated the more severe moisture damage for asphalt mixture. The stripping of FAM components allowed water to more readily penetrate the interior of asphalt mixture specimens with higher surface roughness. The deterioration of coarse aggregate particles affected the load transfer path within the coarse aggregate skeleton of the asphalt mixture when subjected to external mechanical loads. This created weak points in the force chain of asphalt mixture specimens, leading to a reduction in the overall mechanical performance. The FAM component of asphalt mixtures played a direct and critical role in moisture damage resistance. An increase in roughness signified the loss of the FAM component, which facilitated moisture intrusion into the asphalt–aggregate interface and induced stripping. Furthermore, the depletion of the FAM led to dislodging around coarse aggregate particles, altering the pathway of moisture infiltration and exacerbating the overall moisture damage of the asphalt mixture. In this case, the roughness indicators were directly and positively correlated with the degree of moisture damage in the asphalt mixture induced by dynamic water pressure.

The deterioration rates of the FAM component and the coarse aggregate component under erosion by dynamic water pressure differed. The erosion duration of dynamic water pressure conditioning was set at 4000 cycles in this study, and the stripping of the FAM component was more pronounced and significant. This often led to the fact that the concurrent erosion of coarse aggregate particles receives less attention. If the erosion duration of dynamic water pressure was to be further extended, the deterioration of coarse aggregate particles would likely become more evident. Furthermore, the progressive stripping of the FAM component also impacted the coarse aggregate skeleton of the asphalt mixture. As established in the preceding analysis, the spaces surrounding coarse aggregate particles were filled with the FAM component. Continued disintegration of the FAM component allowed for the displacement, rotation, and loosening of coarse aggregate particles, ultimately leading to the collapse of the entire aggregate skeleton. Therefore, the mode of overall moisture damage manifestation in asphalt mixture was governed by the compatibility between the stripping rate of FAM component and the deterioration rate of coarse aggregate particles.

In this study, the asphalt mortar FAM fabricated with SBS-modified asphalt binder demonstrated significantly greater resistance to erosion and stripping by dynamic water pressure compared to that made with base 70# asphalt binder. Although the mechanical strength property of basalt was higher than that of limestone, the porous surface characteristics of basalt rendered its physical and mechanical properties more susceptible to the influence of dynamic water pressure. This could potentially make the coarse aggregate skeleton within basalt-based asphalt mixture relatively more prone to destabilization. Consequently, under the sustained erosion of dynamic water pressure in this study, the asphalt mixture AC-20-Limestone-70# might experience loosening and disintegration of its limestone coarse aggregate skeleton due to the stripping and breakdown of its base asphalt mortar FAM component. For the asphalt mixture SMA-16-Basalt-SBS, fluctuations in the compatibility between the deterioration rate of the basalt aggregate and the stripping rate of the SBS-modified asphalt mortar FAM could lead to an overall reduction in asphalt mixture performance, ultimately dictating the mode of failure. In this case, the deterioration of the basalt aggregate would undoubtedly contribute significantly to the moisture damage of the asphalt mixture. Due to the presence of multiple variables (e.g., water temperature and water pressure magnitude), the comprehensive analysis in this study basically focused on qualitative trends rather than absolute quantitative predictions, while relevant discussions are concluded on the basis of the previous study [23].

## 5. Conclusions

This study explored the moisture damage of asphalt mixture and its components induced by dynamic water pressure using the 3D laser scanning technique. Based on the analysis discussed above, the following conclusions can be obtained:

(1) All roughness indices for limestone-based asphalt mixture increased significantly with a rising water temperature, but these indices for basalt-based samples did not exhibit a clear pattern with dynamic water pressure conditions. Compared to statistical indices, fractal dimension and entropy better captured fluctuating patterns of erosion degree as water pressure magnitude.
(2) The resistance of base asphalt FAM to erosion by dynamic water pressure was weaker than that of SBS-modified asphalt FAM, which resulted in greater changes in roughness indices for limestone-based asphalt mixture.
(3) Limestone’s surface roughness exhibited a clear increasing trend with a rising water temperature, while basalt had relatively smaller change degree in these indices, and their percentage changes showed a greater dependency on variations in water temperature than on water pressure magnitude.
(4) Basalt demonstrated superior mechanical properties compared to limestone, but it possessed a greater abundance of surface micropores or microcracks, which made basalt particles more susceptible to fluctuations under the erosive action of dynamic water pressure.
(5) Compared to water pressure magnitude, water temperature exerted a more significant impact on asphalt mixture and its constituents. The compatibility between the stripping rate of FAM component and the deterioration rate of coarse aggregate governed the macroscopic manifestation of overall moisture damage in asphalt mixture.
(6) Efforts can be made in the future study to link material deterioration and surface roughness features together, which can be helpful to deeply illustrate moisture damage of asphalt materials related to dynamic water pressure.

## Figures and Tables

**Table 1:** Physical properties of asphalt binder.

Items		Units	Base 70# Asphalt Binder		SBS Modified Asphalt Binder	
			Requirements	Results	Requirements	Results
Penetration (25 °C, 100 g, 5 s)		0.1 mm	60–80	64	60–80	74
Softening Point		°C	≥46	48	≥75	77.5
Ductility (5 cm/min)		cm	≥20 (10 °C)	42	≥30 (5 °C)	35
Residue after rolling thin film oven test (163 °C, 85 min)	Mass Loss	%	≤±0.8	−0.177	≤±1.0	−0.204
	Residual Penetration Ratio (25 °C, 100 g, 5 s)	%	≥61	65.4	≥60	65.6
	Residual ductility (5 cm/min)	cm	≥6 (10 °C)	9.8	≥20 (5 °C)	21.2

**Table 2:** Mechanical properties of the asphalt mixture.

Items	Units	AC-20-Limestone-70#		SMA-16-Basalt-SBS	
		Requirements	Results	Requirements	Results
Marshall stability	kN	≥8	11.25	≥6	9.06
Flow value	mm	1.5–4	2.8	–	–
Residual Marshall stability of water immersion	%	≥80	84.7	≥80	84.7
Tensile strength ratio of Freeze–thaw	%	≥75	79.6	≥80	83.8
Rutting dynamic stability	Cycle/mm	≥1000	1539	≥4000	4385

**Table 3:** The proposal of dynamic pore water pressure environment conditioning.

No.	Water Temperature (°C)	Water Pressure Magnitude (MPa)	Duration (Cycles)
1	40	0.4	4000
2	50		
3	60		
4		0.3	
5		0.2	

## Data Availability

The original contributions presented in this study are included in the article. Further inquiries can be directed to the corresponding author.
